# Prevalence of malaria and its environmental and socio-cultural determinants in Tandahimba district council, Mtwara Region, Tanzania

**DOI:** 10.5281/zenodo.21921534

**Published:** 2026-08-13

**Authors:** Sabina J. Ipembe, Hussein Mohamed, Elibariki Mwakapeje

**Affiliations:** 1 Department of Epidemiology and Biostatistics, Muhimbili University of Health and Allied Sciences, PO Box 65015, Dar es Salaam, Tanzania.; 2 Tanzania Field Epidemiology and Laboratory Training Program (TFELTP), Dar es Salaam, Tanzania.; 3 Department of Environmental and Occupational Health, Muhimbili University of Health and Allied Sciences (MUHAS), PO Box 65015, Dar es Salaam, Tanzania.

## Abstract

**Background:**

Malaria remains a major public health and economic burden, especially in sub-Saharan Africa, which accounted for an estimated 94% of the 263 million global malaria cases and 597,000 deaths reported in 2023. In Tanzania, malaria prevalence is estimated at 8%, however regional disparities exist. Mtwara Region reported highest prevalence at 20% in 2022, increasing from 15% in 2017, despite the region's goal to eliminate malaria by 2030. Tandahimba district, in particular, has persistent high transmission despite ongoing control interventions, raising concerns about underlying environmental and socio-cultural factors to malaria transmission. This study aimed to determine the prevalence, environmental and socio-cultural influences linked with malaria cases in the Tandahimba District Council.

**Materials and Methods:**

A mixed-method study using a convergent parallel design was conducted. A multi-stage cluster sampling method was used to select 522 participants. Quantitative data were collected from these participants using structured questionnaires, while qualitative data were obtained through in-depth interviews with Ward Executive Officers and the District Vector Control Officer. Quantitative data were analysed using Stata version 17, applying a modified Poisson regression model to estimate adjusted prevalence ratios (aPRs) with 95% confidence intervals (CIs). Variables with p ≤ 0.05 were considered significant. Qualitative data were analysed thematically using NVivo and integrated with quantitative results to provide a comprehensive understanding.

**Results:**

Among the 522 participants, the mean age was 41 years (±22.5 SD). Maundo ward contributed the highest proportion of participants (24.1%). Environmental factors significantly associated with malaria included earth mud/grass wall materials (aPR = 1.47, 95%CI: 1.19-1.82, p < 0.001) and open eaves (aPR = 1.46, 95%CI: 1.23-1.73, p < 0.001), while metal sheet roofing was protective (aPR = 0.77, 95%CI: 0.69-0.86, p < 0.001). Socio-cultural factors such as travel history (aPR = 1.28, 95%CI: 1.07-1.53, p = 0.006) and staying outdoors at night (aPR = 1.29, 95%CI: 1.09-1.53, p = 0.003) increased malaria risk. Protective factors included bednet use, mosquito repellents, and mosquito sprays.

**Conclusions:**

Malaria prevalence in Tandahimba District is strongly influenced by environmental housing conditions and socio-cultural behaviours. Integrated, community-led interventions addressing housing improvements and preventive practices are essential for effective malaria control.

## Introduction

Malaria remains a major global health concern, causing considerable illness and mortality. In 2023, approximately 263 million malaria cases occurred globally, resulting in an estimated 597,000 deaths. Sub-Saharan Africa bore the greatest burden, accounting for 94% of all cases and approximately 95% of deaths, with children under five years accounting for about 76% of malaria-related deaths [1]. Worldwide, eleven African countries and India combined accounted for approximately 70% of the global malaria burden. Nigeria (26.8%), the Democratic Republic of the Congo (12.3%), Uganda (5.1%), and the United Republic of Tanzania (4.2%) were among the highest-burden countries [1]. While malaria is preventable and treatable, delays in diagnosis and inadequate treatment can result in the disease worsening from mild to severe, which can be life-threatening [2].

In Tanzania, malaria prevalence has declined from 14% in 2015–16 to 8% in 2022, largely due to the implementation of national malaria control strategies. However, this progress has not been uniform across regions [3]. Mtwara Region remains among the areas with the highest malaria burden (20%), and within it, Tandahimba District records an even higher prevalence of 34.4%, surpassing neighbouring districts such as Masasi (4.3%) and Newala (30.4%) [4]. This persistently high prevalence is concerning, given the region's commitment to malaria elimination by 2030 as outlined in the Tanzania National Malaria Strategic Plan (NMSP) [5].

Several factors contributed to variations in malaria risk, including seasonal patterns. The risk is typically highest during the rainy season in tropical countries, such as Tanzania [6]. Other risk factors for malaria among households and individuals include the distance from health facilities [7] and the type of household in which people reside [8,9], the closeness of human settlements to malaria vector breeding sites [10], human behaviours such as evening outdoor activities, time spent outside, care and use of bednets, and health-seeking practices [11,12].

Additionally, socio-demographic factors (age, gender, occupation, and level of education), unsuitable water sources, and insufficient Water, Sanitation, and Hygiene (WASH) conditions are associated with malaria risk [13,14]. Preventive measures against mosquito bites include owning and using Long-Lasting Insecticidal Nets [15,16], residential mobility, and travel [17]. The prevalence of domestic animals near homes [18,19], the abundance of malaria vectors [20] and the timing of mosquito bites [21] have been linked to malaria cases.

Tandahimba district has set a goal to eliminate malaria by 2030. However, progress appears to be slow due to a persistently high malaria prevalence. This issue has contributed to an increase in the Mtwara region's prevalence from 15% in 2017 to 20% in 2022 [5] despite various efforts and actions by the government. Contributing factors are believed to include water supply shortages, the presence of dams and socio-cultural practices such as nighttime social activities.

Although numerous studies in sub-Saharan Africa have reported that poor housing conditions (such as open eaves, thatched roofs, or mud walls) and proximity to mosquito breeding sites contribute to sustained malaria transmission [22], there is limited evidence specifically documenting these associations in Mtwara Region. For example, while research elsewhere has linked building materials and household structure to mosquito entry and increased vector density [22], few studies have systematically examined these environmental factors in Tandahimba District, where malaria prevalence remains disproportionately high. This gap underscores the need for localised evidence to better understand how environmental conditions, particularly housing characteristics, may be influencing malaria persistence in this setting. This study therefore aimed to identify and assess the environmental and socio-cultural factors linked to malaria cases in the Tandahimba district.

## Materials and Methods

A mixed-methods study using a convergent parallel design was conducted from March to May 2025 at the Tandahimba District Council in Mtwara Region, southern Tanzania. The district covers approximately 2,048 km^2^ and is bordered by Mozambique to the south, Newala District Council to the west, Mtwara District Council to the east, and Lindi Region to the north. Tandahimba has a population of about 299,073 people across 89,912 households, with an average household size of 3.3 persons. The area was selected due to its high malaria prevalence (34.4%) compared to other districts in the region.

The study population comprised individuals aged five years and above residing in selected households in six wards of Tandahimba District: Litehu, Luagala, Maundo, Mkwiti, Nambahu, and Ngunja. For the qualitative component, participants included the District Vector Control Officer (DVCO) and Ward Executive Officers (WEOs) from the selected wards.

The sample size was calculated using a single-population proportion formula with a 95% confidence level and an estimated malaria prevalence of 34.4%, based on DHIS2 data. After adjusting for the design effect, the minimum sample size was 522 participants. For the qualitative component, seven (7) key informants were purposively selected from Tandahimba District Council, including six (6) Ward Executive Officers (WEOs), one from each selected ward, and the District Vector Control Officer, who also serves as the Malaria Focal Person.

A multistage cluster sampling technique was used. Three divisions were randomly selected from seven available, and from these, six wards were chosen by simple random sampling. Within each selected ward, three villages were randomly selected, yielding a total of 18 villages. In each selected village, an equal number of households were randomly selected to meet the required number for each ward, determined by population proportional to size. In each selected household, one individual was randomly selected to participate in the study using a lottery method. Only individuals who were present at the time of the visit and gave informed consent were included.

The study included individuals aged five years and above who had resided in Tandahimba District Council for at least 30 days before data collection and provided informed consent or assent (for minors, with guardian consent). For the qualitative component, participants comprised Ward Executive Officers from the selected wards and the District Vector Control Officer. Individuals who self-reported having a fever and took anti-malarial drugs without being diagnosed with malaria at a health facility were excluded from the study.

The outcome variable was self-reported malaria infection diagnosed at a health facility within one month before data collection, categorised as either positive or negative.

The independent variables included demographic factors (age, place of residence, sex, education level, occupation and household size), environmental factors such as the presence of breeding sites, distance of breeding sites from houses, wall building material, open eaves, window screening and roofing material. Socio-cultural factors included travel history, evening outdoor activities, bed-time, type of outdoor activities, presence of a bednet, type of bednet, and use of mosquito repellents, creams, and sprays.

Data were collected through face-to-face interviews using structured questionnaires. Before data collection, informed consent was obtained from all participants, and for those under 18 years, consent was provided by parents or guardians. Local village leaders informed households about the study, and interviews were conducted privately by the principal investigator and trained research assistants.

A structured questionnaire with four sections covering socio-demographic information, self-reported malaria diagnosis within the past 30 days, environmental factors (such as housing conditions and breeding sites), and socio-cultural factors (including travel history, evening outdoor activities, bednet ownership and use, and personal protection practices).

The qualitative component involved semi-structured interviews with Ward Executive Officers from selected wards and the District Vector Control Officer. Separate interview guides were used to explore perceptions and experiences related to environmental and socio-cultural influences on malaria transmission. All interviews were audio-recorded with participants' consent to ensure accurate data capture.

### Data management and analysis

Completed questionnaires were reviewed daily by the Principal Investigator (PI) to ensure accuracy and completeness. Data were double-entered in Excel, cleaned, and exported to Stata version 17 for analysis. Descriptive statistics were used to summarise categorical variables as frequencies and proportions, and continuous variables as means and standard deviations. Univariable modified Poisson regression was used to estimate crude prevalence ratios (cPRs), and variables with p < 0.2 were included in the multivariable model to estimate adjusted prevalence ratios (aPRs) with 95% confidence intervals (CIs). Statistical significance was set at p ≤ 0.05, and model fit was assessed using deviance.

For the qualitative component, in-depth interviews conducted in Kiswahili were audio-recorded with consent, transcribed verbatim, translated into English, and anonymised to ensure confidentiality. Data were analysed using NVivo software through thematic analysis. Open and axial coding were applied to identify relationships and emerging themes related to environmental and socio-cultural factors influencing malaria infection. Findings were presented under thematic categories and supported by participant quotations.

### Ethical approval

Ethical approval for the study was obtained from the Institutional Review Board (IRB) of Muhimbili University of Health and Allied Sciences (MUHAS) (Ref. No. DA.282/298/01.C/2597). Permission to conduct the study was granted by the Mtwara Regional Secretariat (Ref# FA.73/258/01P/219) and Tandahimba District Council. Participation was voluntary, with written informed consent obtained from adults and assent from minors through their parents or guardians. Participants were informed of the study's purpose, procedures, potential risks, and their right to withdraw at any time without penalty. Confidentiality was maintained by anonymising all responses, excluding identifying information, and securely storing data in password-protected files accessible only to the research team.

## Results

### Quantitative results

A total of 522 participants were involved in the study. The mean age of participants was 41 years (±22.52 SD), and the majority of respondents, 419 (80.3%), were in the age group of 18 years and above. Most of the participants originated from Maundo ward 126 (24.1%), and males constituted 275 (52.7%) of all respondents. The majority, 304 (58.2%), had completed primary education, and 225 (43.1%) were farmers. Regarding household size, the majority of participants, 250 (49.7%), lived in households consisting of 5-8 members. Table 1 shows the socio-demographic characteristics of the study population.

**Table 1:** Socio-demographic characteristics of study participants (n = 522).

Variable	n	%
Age group		
<18 years	103	19.7
≥18 years	419	80.3
Place of residence/Wards		
Litehu	61	11.7
Luagala	78	14.9
Maundo	126	24.1
Mkwiti	51	9.8
Nambahu	97	18.6
Ngunja	109	20.9
Sex		
Female	247	47.3
Male	275	52.7
Education level		
No education	83	15.9
Primary	304	58.2
Secondary and above	135	25.9
Occupation		
Unemployed	168	32.2
Farmer	225	43.1
Civil servant/Business	129	24.7
Household size		
1-4	214	41.0
5-8	250	47.9
9-12	58	11.1

### Malaria prevalence and associated factors

The prevalence of self-reported malaria cases, based on diagnoses at health facilities among household members in Tandahimba was 66.6% (95% CI 62.44–70.70%).

Participants who resided in houses with walls constructed from earth, mud or grass materials were 1.47 times more likely to acquire malaria compared to those living in houses made of bricks (aPR = 1.47, 95%CI: 1.19-1.82, p < 0.001). Individuals living in houses with open eaves were 1.46 times more likely to contract malaria than those living in houses with closed eaves (aPR = 1.46, 95%CI: 1.23-1.73, p < 0.001). Metal sheet roofing was protective compared to grass roofing (aPR = 0.77, 95%CI: 0.69–0.86, p < 0.001). Proximity of breeding sites within 5 km was significant in the univariable analysis but not in the adjusted model (aPR = 1.03, 95% CI: 0.86–1.23, p = 0.763). Window screening showed a borderline protective effect (aPR = 0.83, 95%CI: 0.68–1.00, p = 0.051). Regarding socio-cultural factors, participants who travelled outside Tandahimba were 1.28 times more likely to acquire malaria (aPR = 1.28, 95%CI: 1.07–1.53). Bednet users were significantly less likely to contract malaria (aPR = 0.75, 95%CI: 0.60–0.94). Individuals who habitually stayed outdoors at night were 1.29 times more likely to contract malaria (aPR = 1.29, 95%CI: 1.09–1.53). Use of mosquito repellents (aPR = 0.78, 95%CI: 0.65–0.93) and mosquito sprays (aPR = 0.81, 95%CI: 0.69–0.94) were independently protective. The findings of the multivariable analysis are shown in Table 2.

**Table 2:** Multivariable analyses of factors associated with malaria infection in Tandahimba District Council, March - May 2025 (n=522).

Variable	n	Malaria positive n (%)	cPR (95% CI)	p-value	aPR (95% CI)	p-value
**Demographic factors**						
* Place of residence/wards*						
Litehu	61	40 (65.6)	ref	ref	ref	ref
Luagala	78	60 (76.9)	1.18 (0.98-1.34)	0.074	1.03 (0.85-1.26)	0.749
Maundo	126	79 (62.7)	0.96 (0.74-1.23)	0.728	0.88 (0.72-1.07)	0.187
Mkwiti	51	36 (70.6)	1.08 (0.73-1.59)	0.709	1.02 (0.80-1.28)	0.856
Nambahu	97	61 (62.9)	0.96 (0.79-1.17)	0.765	0.83 (0.67-1.02)	0.074
Ngunja	109	72 (66.1)	1.01 (0.81-1.25)	0.948	0.94 (0.74-1.18)	0.586
* Sex*						
Female	247	155 (62.8)	ref	ref	ref	ref
Male	275	193 (70.2)	1.12 (0.98-1.28)	0.105	1.01 (0.91-1.13)	0.861
* Education level*						
No education	83	51 (61.5)	ref	ref	ref	ref
Primary	304	217 (71.4)	1.06 (0.97-1.39)	0.096	1.00 (0.85-1.18)	0.986
Secondary and above	135	80 (59.3)	0.96 (0.73-1.27)	0.795	0.94 (0.79-1.12)	0.480
**Environmental factors**						
* Distance of breeding sites from houses*						
Above 5 kms	86	54 (62.8)	ref	ref	ref	ref
Below 5 kms	345	264 (76.5)	1.21 (1.02-1.46)	0.031	1.03 (0.86-1.23)	0.763
* Building materials*						
Blocks	123	66 (53.7)	ref	ref	ref	ref
Earth muds/grass	399	282 (70.7)	1.31 (1.05-1.66)	0.019	1.47(1.19-1.82)	<0.001
* Open eaves*						
No	127	70 (55.1)	ref	ref	ref	ref
Yes	395	278 (70.4)	1.28 (1.13-1.44)	<0.001	1.46 (1.23-1.73)	<0.001
* Roofing materials*						
Grass	133	115 (86.5)	ref	ref	ref	ref
Metal sheets	389	233 (59.9)	0.69 (0.62-0.77)	<0.001	0.77 (0.69-0.86)	<0.001
* Window screen*						
No	395	277 (70.1)	ref	ref	ref	ref
Yes	127	71 (55.9)	0.79 (0.64-0.99)	0.044	0.83 (0.68-1.00)	0.051
**Social-cultural factors**						
* Travel outside Tandahimba*						
No	265	162 (61.1)	ref	ref	ref	ref
Yes	257	186 (72.4)	1.18 (0.98-1.42)	0.072	1.28 (1.07-1.53)	0.006
* Time of going to bed*						
7:00 to 9:00 pm	141	97 (68.8)	ref	ref	ref	ref
9:01 to 11:00 pm	269	166 (61.7)	0.89 (0.79-1.01)	0.075	1.07 (0.91-1.25)	0.402
11:01 pm to midnight	112	85 (75.9)	1.10 (0.98-1.25)	0.118	1.15 (0.97-1.35)	0.100
* Type of outdoor activities*						
Economic activities	96	69 (71.9)	ref	ref	ref	ref
Others	120	77 (64.2)	0.89 (0.74-1.08)	0.233	0.87 (0.75-1.01)	0.074
Social gathering	99	79 (79.8)	1.11 (0.93-1.32)	0.234	1.06 (0.94-1.20)	0.359
Studies	15	13 (86.7)	1.21 (0.98-1.49)	0.083	1.09 (0.79-1.48)	0.606
* Use of a bednet*						
No	213	174 (81.7)	ref	ref	ref	ref
Yes	224	96 (42.9)	0.52 (0.42-0.65)	<0.001	0.75 (0.60-0.94)	0.012
* Type of bednet*						
ITN	176	97 (55.1)	0.83 (0.70-0.99)	0.034	0.90 (0.78-1.05)	0.173
Non-ITN	261	173 (66.3)	ref	ref	ref	ref
* Staying outdoor habit*						
No	196	110 (56.1)	ref	ref	ref	ref
Yes	326	238 (73.0)	1.30 (1.13-1.49)	<0.001	1.29 (1.09-1.53)	0.003
* Personal prevention*						
Mosquito cream	148	93 (62.8)	ref	ref	ref	ref
Mosquito repellent	131	70 (53.4)	0.85 (0.71-1.01)	0.074	0.78 (0.65-0.93)	0.006
Mosquito spray	133	75 (56.4)	0.97 (0.76-1.06)	0.201	0.81 (0.69-0.94)	0.005
None	110	110 (100)	1.59 (1.35-1.87)	<0.001	1.03 (0.89-1.18)	0.677

## Qualitative results

A total of seven (7) in-depth interviews were conducted with six Ward Executive Officers (WEOs) and the District Vector Control Officer (Table 3). Participants' ages ranged from 30 to 50 years, and 5 (71.4%) had more than two years of experience at their current workstations and held degree-level qualifications. In the qualitative component of the study, interviews were conducted with all six WEOs, and data saturation was reached at that point.

**Table 3:** Demographic characteristics of qualitative participants (n=7).

Variable	Frequency (n=7)	%
Age group		
30-40	4	57.14
41-51	3	42.86
Sex		
Male	6	85.72
Female	1	14.28
Education level		
Certificate	1	14.28
Diploma	1	14.28
Degree	5	71.44
Years of experience at work		
Less than 1 year	2	28.57
2 to 6 Years	5	71.43
Occupation		
Ward Executive Officer	5	85.71
District Malaria Focal Person	1	14.29

The qualitative analysis identified two major themes: environmental factors and socio-cultural factors, along with additional community-level challenges influencing malaria infection in Tandahimba District Council.

### Environmental factors

Based on data collected through in-depth interviews, the study findings revealed that poor water management, mosquito breeding grounds, and inadequate environmental hygiene and sanitation were the key environmental factors associated with malaria infection in Tandahimba DC. Moreover, over half of the participants were concerned about poor sanitation as a major environmental factor contributing to malaria. Participants reported that overgrown shrubs around residences, overflowing pit latrines, and improper waste disposal practices create ideal conditions for mosquitoes to breed.

*“Pit latrines are not well maintained and often overflow, making it easy for mosquitoes to breed.” (Male, 50 years).*

Another participant stated:

*“Other environmental factors contributing to malaria transmission include the presence of dirt and thick bushes around people's residences, which also contribute to mosquito breeding.” (Male, 42 years).*

### Socio-cultural factors

The following socio-cultural factors associated with malaria infection in Tandahimba DC were noted: Cultural norms and practices increasing exposure, beliefs and misconceptions regarding malaria prevention, myths on the use of bednets and government interventions, and misuse and underutilisation of preventive tools, as explained below.

Cultural customs and communal events have been linked to increased malaria risk. Participants reported that cultural ceremonies, such as traditional initiation rites Jando and Unyago, local weddings, frequently expose people, particularly children and women, to mosquitoes because these events are held at night in open or poorly protected environments without any form of protection. Another individual shared:

*"During the Jando and Unyago (traditional weddings) ceremonies, children are isolated in huts for a month without mosquito nets, exposing them to malaria risk." (Male, 50 years).*

### Beliefs and misconceptions regarding malaria prevention

Our study revealed that, despite distribution efforts, actual bednet use remains inconsistent due to cultural beliefs, misconceptions, and a lack of understanding, with participants expressing concern about net misuse or non-use, as evidenced by the following quotes:

*"There's a belief that nets bring bedbugs, so they are often avoided. Some people think nets make men weak or bring illness." (Male, 30 years).*

Other participants expressed this:

*“Even when nets are available, many people don’t use them or use them incorrectly.” (Female, 32 years).*

*“Some people believe mosquito nets cause bad luck or reduce male sexual strength.” (Male, 41 years).*

The study found that during an in-depth interview, participants acknowledged the government's efforts to provide insecticide-treated nets (ITNs), particularly to vulnerable groups such as pregnant women and children. However, they observed that these nets are frequently insufficient for complete household coverage, as stated below:

*“The resources provided, like bednets, are often not enough, and many people are still without protection.” (Male, 42 years).*

*“The last household distribution occurred in 2012. Since then, it seems that the availability of bed nets has decreased. Health centres distribute nets to pregnant women and children under one year old when they come for their first measles vaccination. However, in primary schools, nets are distributed by class levels, not to all students.” (Male, 50 years).*

The study's findings revealed the following challenges besides environmental and socio-cultural factors: resource constraints at the community level, lack of community ownership, and consistent action, all of which affect malaria prevention measures at Tandahimba DC.

Limited household financial capacity was a significant barrier to implementing effective malaria prevention strategies. Participants noted that many families cannot afford to invest in proper sanitation, well coverage, or other preventive measures, often relying on government support for interventions like larvicides. For instance:

*“Families cannot afford to cover wells or buy materials to prevent mosquito breeding.” (Male, 37)*

*“We often wait for government support for things like larvicides.” (Male, 50 years)*

*“Many families cannot afford to cover the wells properly or put in measures to stop mosquito breeding because they don’t have the money.” (Female, 32 years).*

*“There is a lack of consistent use of larvicides, and the community often waits for the government to provide resources.” (Male, 41 years).*

The study revealed a lack of continuous community involvement in malaria control. Many rely entirely on government or health staff and do not accept personal responsibility:

*“The community needs to take ownership and consistently maintain sanitation and water safety. People expect the government to handle everything, but everyone should contribute.” (Male, 30 years).*

*“People are aware that water sources cause mosquito breeding sites and should be covered, but many still ignore the guidance.” (Male, 42 years).*

*“Despite knowing the risks, people continue traditional practices like leaving wells open.” (Male, 50 years).*

The study findings demonstrate a strong convergence between the quantitative and qualitative results, highlighting the multifaceted nature of malaria risk factors in Tandahimba DC. Quantitative data identified housing materials, open eaves, outdoor night activities, travel, and bednet use as significant determinants of malaria risk, aligning with qualitative themes emphasising environmental factors such as poor water management, sanitation, and breeding sites, as well as social and cultural practices that influence exposure. For instance, cultural norms and misconceptions regarding malaria prevention can explain behavioural patterns like outdoor nighttime activities and low bednet coverage observed in the quantitative analysis. Both data types also underscore the barriers posed by limited household net coverage and community resources, reflecting issues such as financial constraints and lack of community ownership, noted qualitatively. While the quantitative results provide measurable associations, the qualitative insights contextualise these findings within community norms, behaviours, and systemic challenges, thus offering a comprehensive understanding of the intertwined environmental, social, and cultural factors influencing malaria transmission in the area.

## Discussion

The study revealed a high period prevalence of self-reported malaria of 66.7% (diagnosed at a health facility within the preceding month) among the general population in Tandahimba DC, Mtwara region. This prevalence aligns with reports from similar endemic areas in Tanzania and sub-Saharan Africa [1,4]. Despite ongoing national malaria control efforts, malaria remains a major public health challenge in the region, but these findings were quite higher than the study done in Muheza District in Tanga region that reported the lowest prevalence of 43.5% [23]. However, another study in the Dar es Salaam region of Tanzania reported the lowest prevalence of 4.5% [24].

The study revealed that participants residing in houses with earth, mud, or grass walls were significantly more likely to contract malaria than those living in houses made of block materials. Poorly constructed houses with porous materials tend to have multiple openings and gaps that facilitate mosquito entry, thereby increasing human-vector contact. This aligns with findings from Tanzania and other sub-Saharan African countries, where studies have consistently demonstrated that sub-standard housing materials are associated with increased indoor vector density and higher malaria prevalence [8,9]. According to the World Malaria Report 2024 [1], housing improvements are recognised as part of integrated vector management (IVM) strategies, as better housing structures can significantly reduce vector access. Similarly, Tanzania's Integrated Vector Management Guidelines emphasise the importance of environmental modification and structural improvements, including housing design, to limit mosquito breeding and resting sites. These national guidelines are echoed by the results of this study and others, such as the study done in Ruhuha in the East of Rwanda, which found that lower housing quality (made of mud, unburnt brick) was linked with malaria cases [1,3,25].

The present study found that individuals residing in houses with open eaves were significantly associated with malaria cases compared to those living in houses with closed eaves. Open eaves may facilitate mosquito entry into houses, increasing human-vector contact during peak mosquito biting hours. Qualitative findings further revealed that participants living in such houses frequently experienced high mosquito presence indoors, particularly during the rainy season. Similar findings have been reported in studies conducted in Lake Tana, Muheza District in Tanzania, Debre District in Ethiopia, and a scoping review in sub-Saharan Africa, where open eaves and wall openings increased malaria risk [18,22,25,26]. However, studies conducted in North-West Tanzania and Dar es Salaam reported no significant association between open eaves and malaria cases [24]. These differences may reflect variations in malaria transmission intensity, vector density and behaviour, housing conditions, and local ecological characteristics across settings [27].

The differences between Tandahimba and areas such as Dar es Salaam and North-West Tanzania may be due to variations in malaria transmission intensity, environmental conditions, housing structures, outdoor behaviours, and socioeconomic factors. Tandahimba is a high-transmission rural setting, while Dar es Salaam has lower transmission due to urbanisation and improved housing. These differences support Tanzania's subnational malaria stratification approach for targeted interventions.

Individuals living in houses with metal sheet roofing were less likely to experience malaria compared to those living under grass-thatched roofs. This finding aligns with growing evidence that housing structure significantly influences malaria transmission risk due to its impact on mosquito entry, indoor resting behaviour and survivability of malaria vectors [22]. Metal roofs offer greater protection against mosquito entry due to their tighter construction and fewer entry points compared to traditional grass-thatched roofs. In contrast, grass roofing tends to have multiple openings and gaps, creating easy entry and resting spaces for mosquitoes.

Similar findings have been documented in rural malaria-endemic settings in Tanzania, Burkina Faso, and other parts of sub-Saharan Africa, where poorly constructed traditional houses were associated with higher indoor mosquito density and malaria risk [8,11,28]. The protective effect of metal roofing may be related to reduced mosquito entry and less favourable indoor resting conditions for malaria vectors. All these findings differed from those of the study conducted in Dar es Salaam, Tanzania, and Lake Tana, Ethiopia, which found that the type of roof, whether metal or thatched, was not associated with malaria cases [18,24].

Our study underscores the significance of housing structures as critical determinants of malaria and highlight the importance of integrating housing improvements into malaria elimination strategies, particularly in Tandahimba District, where traditional housing structures are still common.

Individuals who travelled outside Tandahimba District were more likely to contract malaria compared to those who did not travel. Travel may increase exposure to different malaria transmission environments, particularly when individuals move between high-transmission rural areas. However, this study did not determine the proportion of imported versus locally acquired malaria cases. Therefore, the findings should be interpreted cautiously. Future studies incorporating travel surveillance and parasite characterisation may help distinguish imported infections from local transmission dynamics. A study in northern Namibia, Zanzibar and Rwanda have documented similar dynamics and identified travel as a significant predictor of malaria cases detected through reactive case detection in Zanzibar, suggesting that control efforts must include mobile populations and in Rwanda spatial modelling was used to show that imported cases fuelled local outbreaks even in low-transmission zones. In the context of Tandahimba, where people often travel for economic or social reasons, these patterns are relevant [25,29,30]. Our findings differed from those of the study conducted in Ethiopia, northwestern Tanzania, lower Moshi, and Dar es Salaam, which found that travel history is not a significant factor for malaria [7,17,26].

The current study found that participants in the general study population who reported sleeping under bednets were significantly less likely to be infected with malaria than those who did not use nets. This supports ongoing national malaria prevention strategies, including LLIN distribution through antenatal clinics, postnatal services, school-based campaigns, and community distribution programs.

The Tanzania Demographic and Health Survey and Malaria Indicator Survey [TDHS-MIS, 2022] similarly reported lower malaria prevalence among households consistently using LLINs, especially in regions with a high malaria burden. This aligns with WHO's World Malaria Report [1] and the Global Malaria Programme's Framework for Malaria Elimination, both of which emphasise LLINs as an effective vector control tool that has substantially contributed to the global reduction in malaria incidence over the past two decades [1,2]. Moreover, findings from studies conducted in and across other endemic regions, such as Ethiopia and Nigeria, found that consistent net usage reduces the risk of malaria infection. These studies have demonstrated reduced vector density and human-vector contact in households where LLINs are properly used and maintained [8,17,26].

Individuals who reported staying outdoors at night were significantly more likely to contract malaria compared to those who remained indoors. These findings were similar to the study done in Dembiya District in Ethiopia and a meta-analysis done in sub-Saharan Africa that individuals with frequent outdoor staying habits were more vulnerable than those with no or limited outdoor staying habit. This can be due to increased individual exposure to the malaria parasite [17,31]. Contrasting findings, however, have been documented in studies where outdoor behaviour was not significantly associated with malaria transmission. For instance, in the Lower Moshi region of Northern Tanzania and Burkina Faso, researchers found that other factors, rather than staying outdoors, habit and evening outdoor activities were significantly associated with malaria cases [6,7,12,32].

Although outdoor nighttime behaviour was strongly associated with malaria infection in Tandahimba, the strength of this association may vary across settings depending on local vector ecology, malaria transmission intensity, and human behavioural patterns. Therefore, context-specific malaria prevention strategies remain essential in high-transmission rural settings such as Tandahimba. Qualitative findings revealed that common nighttime outdoor activities included social gatherings, small-business activities, studying, ceremonies, and household interactions that occurred between evening and late-night hours. These periods overlap with peak biting times of malaria vectors, thereby increasing exposure to mosquito bites and malaria infection.

Individuals who used mosquito repellents and mosquito sprays were significantly less likely to contract malaria compared to those who did not, demonstrating the effectiveness of additional personal protection methods. Similar evidence from studies in Indonesia and Mozambique supports the use of repellents as complementary strategies; also, these findings align with WHO's recommendations for integrated vector management, especially in areas with high residual transmission [2,14,27]. Our finding was different from the study done in Burkina Faso, Mozambique and Dar es salaam which reported that despite the high burden of malaria, the use of personal protection measures such as sprays and repellents was not significantly associated with reduced malaria cases [14,24,28].

The findings have significant public health implications for malaria control in high-burden districts, such as Tandahimba. First, environmental risk factors such as poor housing highlight the need for structural interventions beyond commodity distribution, such as promoting durable housing materials and improved water management. Socio-cultural practices, including extended outdoor evening activities and travel to endemic areas, demonstrate the importance of tailoring health education to local behaviours. The protective role of bednets, repellents and sprays reinforces the need to sustain high coverage while addressing barriers to consistent use. By integrating environmental management with culturally responsive health education, malaria control strategies can be more effective and sustainable in high-prevalence districts.

A qualitative result revealed poor water management, specifically uncovered underground rainwater storage, other facilities and stagnant water act as breeding sites for mosquitoes. This is consistent with findings from studies which demonstrated that poorly maintained waterbodies around large dams significantly increased malaria risk in sub-Saharan Africa [10]. Additionally, inadequate sanitation, such as overgrown vegetation and improperly managed latrines, was identified as a malaria risk, reinforcing observations on environmental and sociodemographic factors associated with malaria in Congo [13], where poor household hygiene was associated with increased malaria burden. The qualitative findings from Tandahimba reflect a recurring challenge in low-resource settings, where environmental management is often neglected due to financial constraints.

While using a bednet offers protection, this underscores that usage and not ownership is crucial, echoing lessons from the Tanzania demographic health survey and malaria indicator survey [4,6]. Although government ITN distribution was acknowledged, participants from the in-depth interview highlighted insufficient net supply as a persistent problem, especially for larger households. This aligns with findings from north-western Tanzania on risk factors for malaria infection prevalence and household vector density between mass distribution campaigns for LLINs, which reported coverage gaps in ITN distribution campaigns in north-western Tanzania, resulting in partial protection within households. Also, other studies argue, equitable access to vector control tools must be coupled with behavioural interventions to ensure effective use [8,31].

Participants reported several barriers to consistent net use, including beliefs that nets attract bedbugs, a concern that has been documented in the literature [33] as well as beliefs that nets cause bad luck or reduce male sexual strength. While the association between ITNs and bedbugs has scientific support, the latter beliefs reflect cultural misconceptions that continue to hinder uptake. These findings underscore the need for contextually sensitive behaviour change communication that acknowledges documented concerns while addressing unfounded fears. This demonstrates that despite broad awareness campaigns, behaviour change remains elusive, particularly when prevention efforts fail to address deep-rooted cultural norms [15,34]. Qualitative findings also revealed that cultural practices such as night-time ceremonies like Jando and Unyago were described as contributing to mosquito exposure. These observations mirror findings in south-eastern Tanzania, which linked social gatherings and outdoor nighttime behaviour to increased mosquito bites and malaria transmission [11].

Qualitative results revealed additional findings that emphasised that financial limitations hindered community efforts to cover wells or maintain environmental cleanliness. This was also reported in a meta-analysis on socioeconomic development as an intervention against malaria, which showed that economic deprivation consistently increases malaria vulnerability. Moreover, while health education campaigns have raised awareness, participants stressed the disconnect between knowledge and practice. Similar patterns were observed in Rwanda and Indonesia, where behavioural reluctance persisted even in the face of adequate knowledge and interventions [21,35]. The lack of community ownership emerged as a central barrier to sustained malaria control. Participants reported a dependence on government interventions, with limited grassroots initiative. Studies emphasised the importance of community participation in vector control, showing that locally-led larviciding campaigns yielded stronger outcomes in southern Tanzania, and other studies proposed a citizen science framework to strengthen community engagement in malaria prevention in Rwanda, which could be adapted to the context in the study area [36,37].

Our findings have significant public health implications for malaria control in high-burden districts, such as Tandahimba. First, environmental risk factors such as poor housing highlight the need for structural interventions beyond commodity distribution, such as promoting durable housing materials and improved water management. Socio-cultural practices, including extended outdoor evening activities and travel to endemic areas, demonstrate the importance of tailoring health education to local behaviours. The protective role of bednets, repellents and sprays reinforces the need to sustain high coverage while addressing barriers to consistent use. By integrating environmental management with culturally responsive health education, malaria control strategies can be more effective and sustainable in high-prevalence districts.

### Study limitations

This study faced potential biases that could have influenced the findings. Selection bias might have occurred if households that were absent during data collection differed systematically from those included. Additionally, information bias is likely since malaria prevalence was based on participants' self-reported diagnoses within the past one month, which could have led to overestimations of true prevalence due to misclassification or recall errors. To reduce these biases, the study focused on recent malaria episodes within the past month, minimising memory distortion, and employed clear, simple questions to enhance response accuracy. Although triangulating with qualitative data helped strengthen the overall validity of the findings, these limitations should still be taken into account when interpreting the results.

## Conclusions

Our findings suggest that despite ongoing malaria control interventions, persistent environmental and behavioural factors continue to contribute to malaria transmission in high-burden settings like Tandahimba. We highlight the importance of integrating environmental management, improved housing, and culturally responsive malaria prevention strategies into existing malaria control programmes. There is also a need for further operational research and pilot interventions to assess the feasibility and effectiveness of housing improvements, including the promotion of durable building materials such as bricks and metal roofs, and the installation of closed eaves to reduce mosquito entry. However, additional longitudinal and entomological studies are needed to better establish causal pathways and quantify the contribution of these factors to malaria transmission.
